# Peer Review, Program Officers and Science Funding

**DOI:** 10.1371/journal.pone.0018680

**Published:** 2011-04-12

**Authors:** Paul J. Roebber, David M. Schultz

**Affiliations:** 1 Atmospheric Science Group, Department of Mathematical Sciences, University of Wisconsin at Milwaukee, Milwaukee, Wisconsin, United States of America; 2 Centre for Atmospheric Science, School of Earth, Atmospheric and Environmental Sciences, University of Manchester, Manchester, United Kingdom; 3 Division of Atmospheric Sciences, Department of Physics, University of Helsinki, Helsinki, Finland; 4 Finnish Meteorological Institute, Helsinki, Finland; Science and Technology Facilities Council, United Kingdom

## Abstract

Increased competition for research funding has led to growth in proposal submissions and lower funding-success rates. An agent-based model of the funding cycle, accounting for variations in program officer and reviewer behaviors, for a range of funding rates, is used to assess the efficiency of different proposal-submission strategies. Program officers who use more reviewers and require consensus can improve the chances of scientists submitting fewer proposals. Selfish or negligent reviewers reduce the effectiveness of submitting more proposals, but have less influence as available funding declines. Policies designed to decrease proposal submissions reduce reviewer workload, but can lower the quality of funded proposals. When available funding falls below 10–15% in this model, the most effective strategy for scientists to maintain funding is to submit many proposals.

## Introduction

At the U.S. National Science Foundation (NSF), 96% of funds are awarded through a competitive merit review [1]. Proposal success rates across that agency, however, have decreased from 27% in 2002 to 20–22% in 2004–2008 [1]. As more scientists compete for increasingly limited government funding, scientists may feel pressure to submit more grant proposals (e.g., the number of proposals received at NSF has increased 40% since 2001) [1]. These difficult conditions are familiar to practicing scientists and are mirrored at agencies throughout the U.S. and internationally [2].

This increased competition for limited resources has established the scientific equivalent of the famous problem in game theory known as the Prisoner's Dilemma [3]. In the Scientist's Dilemma [4], [5], the rational strategy for maximizing research funding is to submit as many proposals as possible, yet, as in the classic form of the Prisoner's Dilemma, this strategy is less optimal than cooperation. If scientists agreed to limit the number of proposals that they submitted or were required to limit that number [3], they would not incur the substantial time penalty of writing and reviewing many proposals, but might retain equivalent chances for funding. The costs of excessive competition extend beyond the individual to science and society as a whole. The extra work resulting from increasing numbers of proposals does not increase the total pool of research money available to scientists but limits their ability to conduct research, as well as to teach and mentor students.

Under what conditions is submitting as many proposals as possible an inefficient strategy? How do selfish or negligent reviewers affect the peer-review process [6]–[8]? How is funding the highest-quality research ensured? Above all, how do the rules of the funding agency and the program officers' approach to decision-making affect the community?

## Methods

To explore these questions, we employ an agent-based model of peer review and funding, modified from [9]. Traditional quantitative models use differential equations to represent continuous flows. Agent-based models, in contrast, represent complex systems through the interaction of discrete entities (agents) in which the system behavior emerges through the aggregate effects of these agent interactions. In the model of the peer review system, the agents are (1) the scientists who write proposals, (2) the other scientists, taken from that same pool, who review those proposals, and (3) the funding agency program officers who make funding decisions based in part upon those reviews. A set of rules governs the response of these agents to various conditions (e.g., the level of available funding).

**Figure 1:**
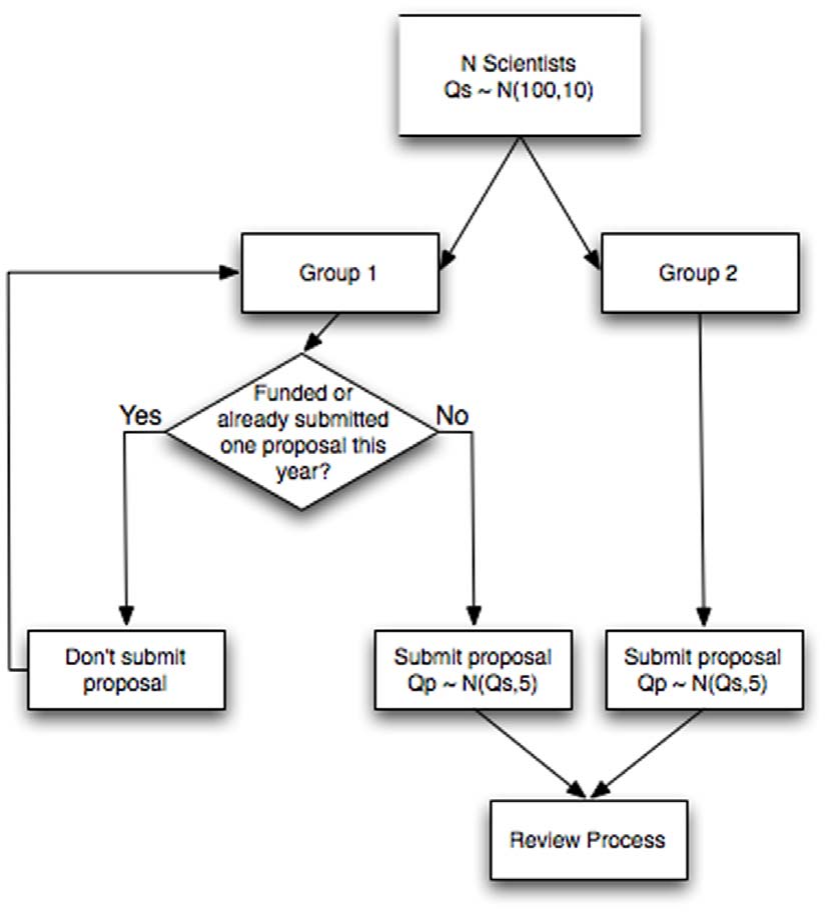
Proposal generation and submission process. Proposal generation by *N* scientists, of quality *Qs*, drawn from a normal population of mean 100 and standard deviation 10. Proposals are of quality *Qp*, drawn from a normal population of mean *Qs* and standard deviation 5. Arrows indicate the flow of decisions through the proposal submission process.

In this system, proposal development is modeled as follows (Fig. 1). A community of *N* scientists is composed of two evenly sized groups. Group 1 (G1) produces one proposal every two time units (split randomly between those times), whereas group 2 (G2) produces a proposal every time unit. We set a time unit to six months, award length to three years, and all grants to equal value. G1 scientists who obtain funding do not submit new proposals until the final year of the grant. In contrast, G2 scientists continue to submit grant proposals regardless of their funding status. We assume that the quality of an individual scientist (*Qs*) follows a normal distribution with mean 100 and standard deviation 10. Each scientist produces proposals of variable quality *Qp*, which are drawn from a normal distribution with mean *Qs* and standard deviation 5. These characteristics are identical in both groups.

**Figure 2 pone-0018680-g002:**
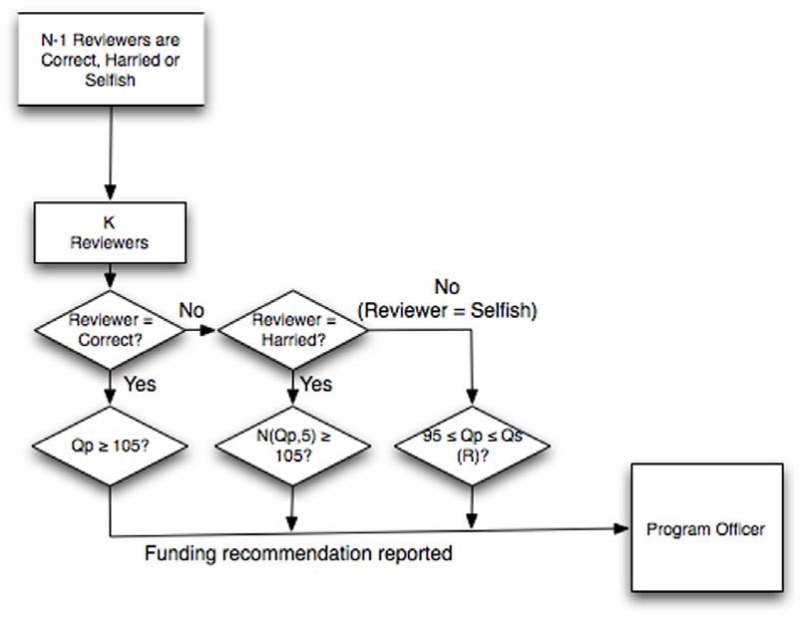
Proposal peer-review process. Proposal review process, accomplished by *K* scientists, randomly selected from the set of *N-1* scientists (excluding the scientist who submitted the proposal under consideration). *Qs (R)* is the scientific quality of the reviewer. Arrows indicate the flow of decisions through the proposal review process.

Proposal review is then modeled (Fig. 2). Each proposal is reviewed by *K* independent reviewers, randomly chosen by the program officer of the funding agency from the *N*–1 available scientists (that is, excluding the proposal author). No limits are placed on the number of reviews that a scientist can conduct. The reviewers recommend to the program officer either to fund or to decline. Each reviewer is one of three types: correct, harried or selfish. The correct reviewer recommends funding only for high-quality proposals, defined as those exceeding a minimum threshold (i.e., in the top 16% of all proposals or at least one standard deviation above the mean). The harried reviewer behaves like the correct reviewer, but assesses proposal quality imperfectly. This assessment is modeled as a normal distribution with mean *Qp* and standard deviation 5; that is, the assessments are correct in the mean but not necessarily on a case-by-case basis. Finally, selfish reviewers recommend declining a proposal that is either superior to their own work or below a minimum quality (i.e., if *Qp* is greater than the *Qs* of the reviewer or less than 90% of the defined minimum threshold). Thus, a low-quality scientist who is a selfish reviewer might recommend rejection of nearly all proposals considered, whereas a very high-quality scientist but selfish reviewer would recommend rejection of relatively few high-quality proposals.

**Figure 3:**
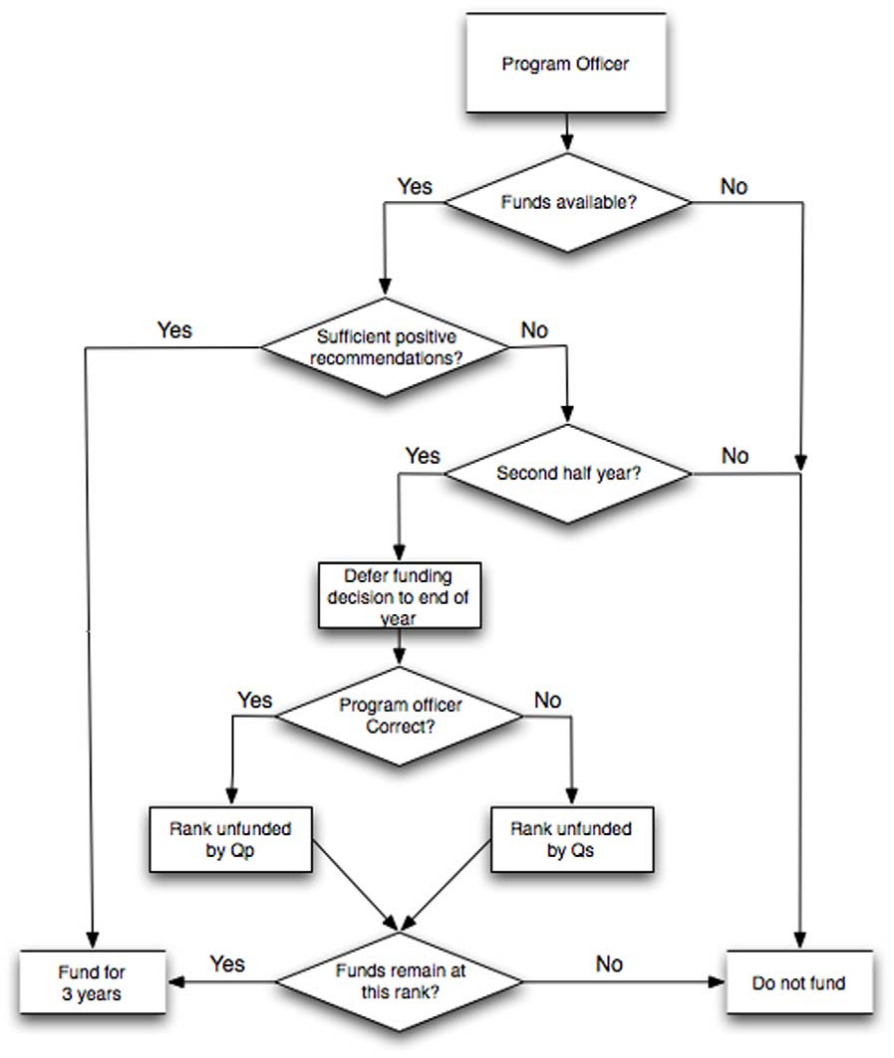
Program officer decision-making process. Proposal funding decision process, accomplished by the funding agency program officer. Arrows indicate the flow of decisions through the funding decision process.

Next, the funding decision is modeled (Fig. 3). The annual budget for program officers is represented by a specified target funding rate. In the first half-year, the program officer uses that rate to estimate the number of fundable proposals and then funds proposals with unanimously positive recommendations up to half that limit; after which, all proposals are declined regardless of quality. In the second half-year, this process is repeated. If there is a surplus of funds at the end of the year, then the highest-rated unfunded proposals of the last six months are reconsidered until the target rate is reached.

The decision to fund reconsidered proposals is made according to one of two program-officer types: correct or reputation-based. The correct program officer has perfect knowledge of *Qp* and thus can precisely rank proposals. The reputation-based program officer is a correct program officer who substitutes quality of the scientist *Qs* for *Qp* in the rankings. The model logic detailed in Figures 1–3 was implemented in FORTRAN, with agent properties developed using random number generators over a long set of simulations (here, 2000 time steps).

## Results

To start, we form a perfect scenario in which all reviewers and the program officer are correct, and for which there are no external funding limits (Table 1, case a). In this situation, the highest-quality proposals are properly judged, and 30.2% of all proposals are funded. Although the funded-proposal quality for the two groups is similar, owing to their divergent proposal-submission strategies, G2 obtains 76.9% of the available funding. Furthermore, because high-quality G1 scientists tend to get funded and drop out of the funding cycle for three years, the medium- and low-quality G1 scientists are left to compete against all the persistent G2 scientists. Consequently, G1's success rate of 21.3% is considerably lower than G2's 34.6%.

**Table 1:** Agent-based model experimental results.

Experiment	Group 1		Group 2		Group 2 hare of Funding (%)
	Scientist unding Success (%)	Average Quality of Funded Proposals	Scientist unding Success (%)	Average Quality of Funded Proposals	
**a. Perfect**	21.3	111.6	34.6	112.1	76.9
**b. Baseline**	25.2	115.0	9.9	112.8	44.8
**c. More Selective (Top 2%)**	34.8	111.6	4.5	116.4	19.8
**d. Positive Feedback**	17.8	117.6	13.5	113.8	60.6
**e. Negative Feedback**	33.9	109.7	5.6	112.0	25.1
**f. Limiting G2 Scientists to One Grant**	29.4	109.1	4.8	112.5	19.1
**g. Cooling-Off Period**	12.5	115.7	17.4	113.0	57.7

Results are stratified according to the two scientist groups. Shown for each group are the scientist funding success and the average quality of funded proposals. The group 2 share of the available funds is also shown.

Next, we form a baseline case with a mix of reviewers (60% correct, 20% harried and 20% selfish) and a target funding rate of 15% (Table 1, case b). This target rate is half that of the perfect scenario, so the participants feel some funding pressure. Funded-proposal quality is higher in both groups, but is several points higher in G1 than in G2, with G2 receiving only 45% of available funding despite submitting more than twice as many proposals. Owing to increased proposal submissions from both groups in the tighter funding climate, the average number of reviews conducted per scientist increases by 13% over the perfect scenario. How do these changes, including a nearly 33% swing in funding share between the two groups, occur?

**Figure 4:**
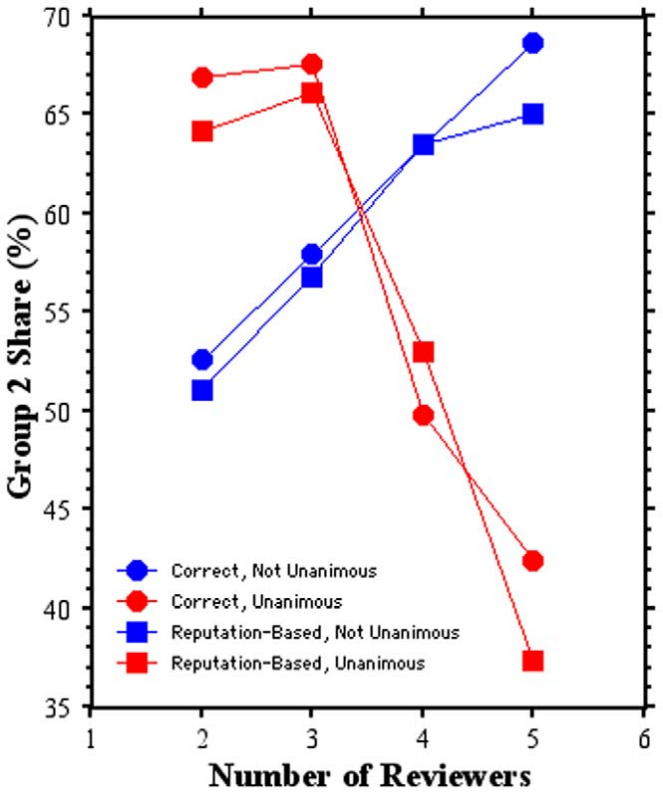
Group 2 share (%) of available funding based on the review process. Shown for the two types of program officers (correct, circles; reputation-based, squares), using from two to five external reviewers and forming an initial funding decision based on unanimous (red symbols) or non-unanimous recommendations (blue symbols).

The decisions by the program officer (number of reviewers and program officer type) determine the outcome of many proposals at NSF, where about 25% of funding decisions are made by the program officer contrary to the reviewer recommendations [10], and in the model (Fig. 4). Generally, G2 scientists command an increasing share of the funding as the number of reviewers increase, except in two situations (Fig. 4). If correct or reputation-based program officers require unanimity among four or more reviewers, then the G2 share of funding drops considerably compared to what would have happened had the program officer selected fewer reviewers or not required unanimity in the reviewer recommendations. If a program officer were more selective by raising the minimum threshold to the top 2% (at least two standard deviations above the mean), funding for G1 increases because relatively more G2 proposals are declined (Table 1, case c).

**Figure 5:**
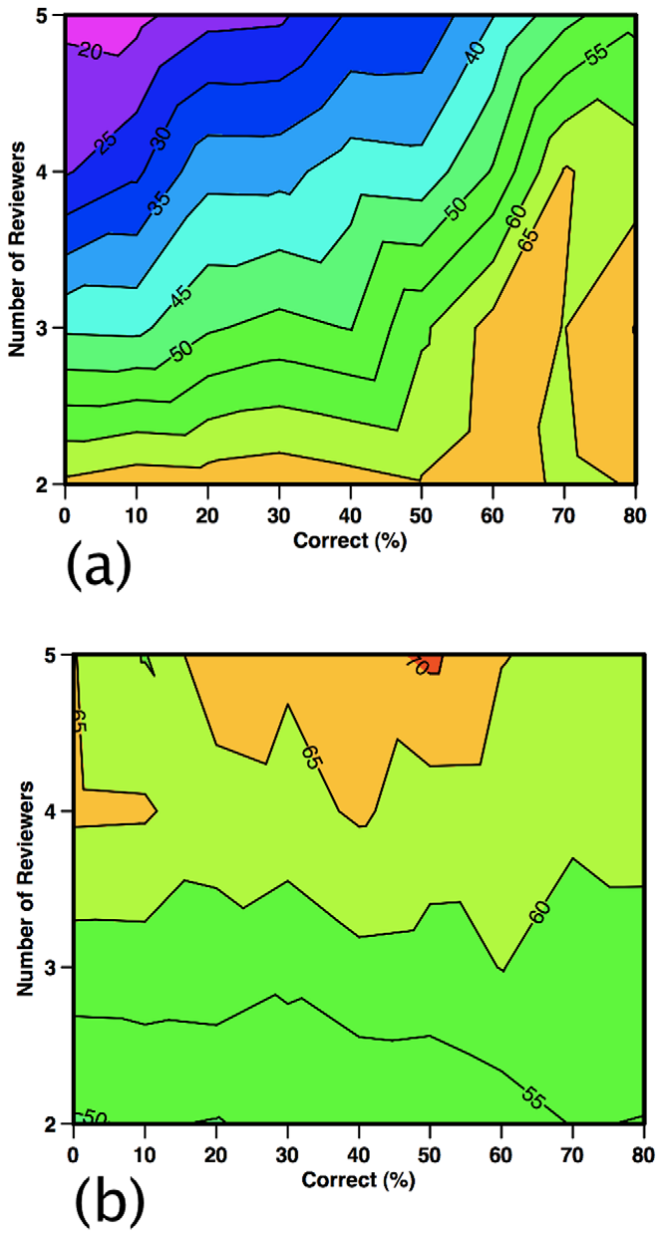
Group 2 share (%) of available funding based on the reviewers. Shown as a function of the number of reviewers and the reviewers who are of the correct type (%). Selfish reviewers are fixed at 20% and the remainder are harried reviewers. The program officer is correct and initial funding decisions are made using (a) unanimous reviewer recommendations or (b) allowing one negative recommendation.

**Figure 6:**
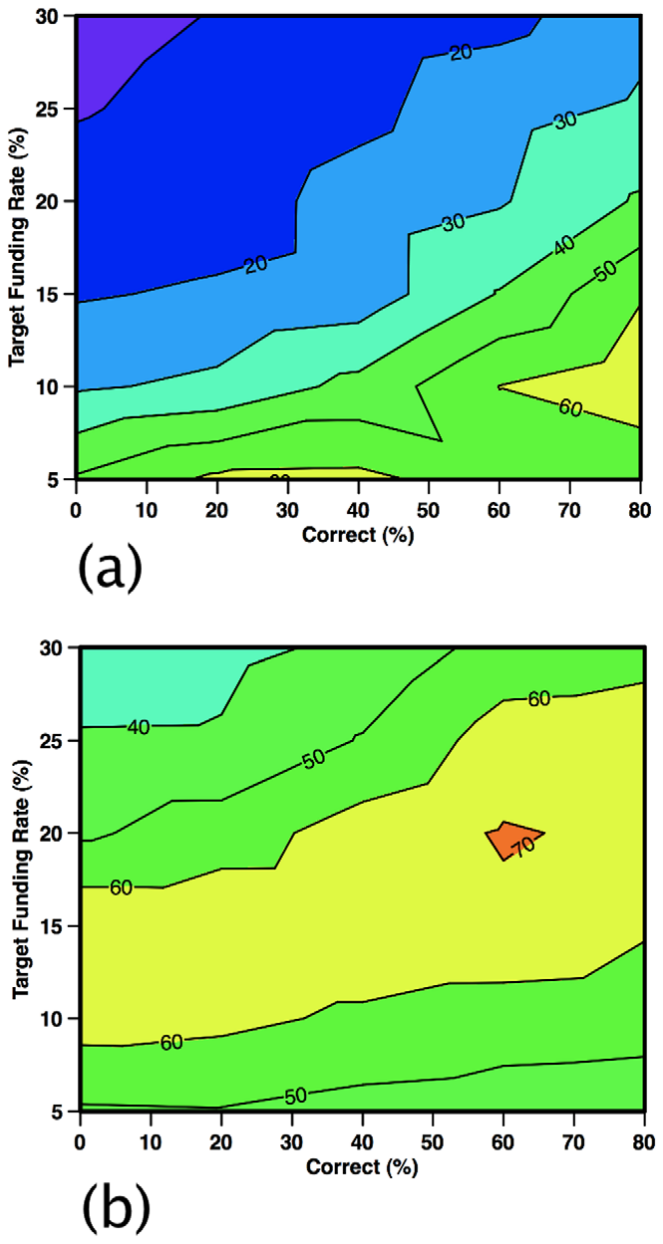
Group 2 share (%) of available funding based on target funding rate. Shown as a function of the target funding rate (%) and the reviewers who are of the correct type (%). Selfish reviewers are fixed at 20% and the remainder are harried reviewers. The program officer is correct and initial funding decisions are made using (a) five unanimous reviewer recommendations or (b) allowing one negative recommendation.

Counterintuitively, imperfections in the review process are also key. The presence of non-correct reviewers retards the effectiveness of the G2 strategy, especially when program officers use more reviewers and require unanimity (Fig. 5a). If, however, program officers fund proposals receiving one decline recommendation (Fig. 5b), then the success rate of G2 is nearly insensitive to the percentage of non-correct reviewers and increases slightly with more reviewers. As funding diminishes, G2 receives an increasing share of the resources under a program officer requiring unanimity (Fig. 6a). In contrast, for a program manager funding proposals with one decline recommendation, G2's share is less sensitive to the percentage of non-correct reviewers and reaches a maximum around 10–20% target funding rates (Fig. 6b).

The quality of funded proposals is also sensitive to the reviewer mix for G1 and G2, as suggested in actual funding data [6], [11] and in models [9], [12]. Although not explicitly modeled, the effect of increasing reviewer load can be discerned from these results—as correct reviewers are converted into harried ones by this load, the funding share of G2 declines (Fig. 6) and the quality of the funded proposals slightly improves. This feedback suggests that scientists, were they aware of this effect, would write fewer grant proposals to maximize their efficiency. A critical caveat, however, is that this feedback loses effectiveness once funding rates decline below about 7% (Fig. 6).

So far, we have assumed that no relationship exists between the number of proposals submitted and their quality. However, a positive feedback might exist if G2 scientists submit many proposals owing to the highly capable and productive research groups that they have assembled. Further, individual proposals might benefit from peer review and be improved for subsequent submission. On the other hand, a negative feedback might exist if G2 scientists simply churn out mediocre-quality proposals. Not surprisingly, positive feedbacks (defined as a 5-point boost in *Qp* for G2) increase G2's share of the funding, whereas negative feedbacks (a 5-point drop in *Qp* for G2) do the opposite (Table 1, cases d and e). Interestingly, the quality of funded proposals by G1 scientists also increases for positive feedbacks and decreases for negative feedbacks, as only the success of the G1 scientists depends upon the level of the competition from G2. Most likely positive, neutral and negative feedbacks on rapid-fire proposal writers all exist in any mix of individuals, and their relative proportions ultimately would determine their importance to funding success.

What if the behaviors of G1 or G2 change? If G1 scientists are allowed to pursue additional grants regardless of current funding status, only minor changes from the baseline occur (not shown), whereas limiting G2 scientists to one funded grant substantially reduces G2's share of funding and success rate (Table 1, case f). In this latter scenario, the reviewer load decreases by 22% but the quality of G1's funded grants declines, owing to the reduced competition from G2.

Funding agencies also have tried to decrease the number of proposal submissions through other means. For example, the U.K. 's Engineering and Physical Sciences Research Council (EPSRC) bars proposal submissions from scientists for 12 months if, in the preceding two years, they have had at least three proposals ranked in the bottom half of a funding prioritization list or were rejected by a panel review and have an overall success rate of less than 25% [2], [13]. A simulation incorporating this "cooling-off period" reduces the proposal-review burden by 34% relative to the baseline, largely by equalizing G1 and G2 submission rates. Collateral effects include a halving of funding success for G1 scientists with an increase in the G2 funding share to 57.7%, owing to the differential removal of lower-quality G2 proposal writers (Table 1, case g).

## Discussion

Our results emphasize the importance of the program officer's approach and funding agency's rules, which can vary substantially across divisions within the same agency and over time [8,14,15]. This is consistent with recent research that suggests that optimal decision-making is a complex interaction of factors including the number of judges, the accuracy of those judges, and the decision rules used [12]. Program officers who use more reviewers (e.g., for NSF mail reviews, the FY 2009 average is more than four) [1] and require unanimity limit the efficiency of G2's many-proposal strategy. Program officers who base decisions on the quality of the scientist fund higher-quality proposals and increase the success rate of individual scientists.

Highly capable research clusters may profitably and efficiently choose to submit many proposals, which may or may not be tolerable from the perspectives of science and society because of the increased proposal burden and possibly lower-quality funded research [11]. Reducing the proposal burden by the cooling-off period or by limiting grants to one award per investigator appear to achieve their primary aim, but can produce collateral effects on funded-proposal quality and the success rate of individuals. The negative feedback from proposal churning may be sufficiently corrective such that additional constraints may be unnecessary. Once available funding falls below 10–15% in our model, however, submitting many proposals, despite the tax that this represents on both individuals and their scientific communities, appears to be the only recourse if the goal is to maintain research funding.
